# Use of Patient-Specific Information for Randomization in Clinical Research: A Randomized Trial

**DOI:** 10.1016/j.acepjo.2025.100215

**Published:** 2025-06-30

**Authors:** Jakub Furmaga, Jonathan Reeder, Robert Turer, Ellen O’Connell, Bhaskar Thakur, Samuel McDonald

**Affiliations:** 1 Department of Emergency Medicine, University of Texas Southwestern Medical Center, Dallas, Texas, USA; 2 Department of Occupational Health, University of Texas Southwestern Medical Center, Dallas, Texas, USA; 3 Clinical Informatics Center, University of Texas Southwestern Medical Center, Dallas, Texas, USA

**Keywords:** clinical research, randomization method, randomization performance, electronic medical records, pragmatic trial, A/A testing, cryptographic standards

## Abstract

**Objectives:**

Technology companies conduct tens of thousands of automated experiments each year using A/B testing to optimize their products. Their methods rely on field experiments incorporated into customer-facing products. In medicine, pragmatic trials do the same by performing experiments alongside usual care. This study aims to assess the effectiveness of using patient-specific information for study group randomizations.

**Methods:**

We developed an electronic medical records-based randomization method (EMRRM) that used parity of encounter identification (ID) and patient ID numbers to separate patients into 2 random groups. This method was retrospectively applied to patients in the outpatient, inpatient, and emergency department settings. To assess the randomness of group assignments, we used the National Institute of Standards and Technology's Special Publication 800-22 (NIST) statistical package for randomness testing, and A/A testing for group similarities. The main outcome measure was the EMRRM’s ability to perform group randomization as assessed by NIST and A/A test results.

**Results:**

For each of the clinical settings, encounter ID and patient ID methods passed either 10 or 11 out of 11 applicable NIST tests. On A/A testing, both methods successfully randomized patients into similar groups with equivalent demographics, laboratory tests, lengths of stay, and hospital admission rates.

**Conclusions:**

This study validated the use of encounter ID and patient ID-based EMRRMs in pragmatic studies. Patient information-based EMRRM can be used for automatic random study group assignments in pragmatic trials.

**The Bottom Line**

Pragmatic trials work best when a reliable randomization method is incorporated directly into existing electronic medical record systems. Most randomization platforms, however, cannot do that. Our study examined an electronic medical records-based randomization method that used parity of encounter identification (ID) and patient ID numbers to separate patients into 2 random groups. This randomization method passed 10 out of 11 applicable NIST 800-22 statistical package tests for randomness testing and divided populations into very similar groups as assessed using A/A testing. This study validates encounter ID and patient ID-based electronic medical records-based randomization methods for automatic random study group assignment.

## 1 Introduction

### 1.2 Background

Large technology companies, such as Amazon, Microsoft, Facebook, and Google, conduct tens of thousands of experiments each year to optimize their services.^1^ These trials are so well incorporated into customer-facing products that most people do not notice them. This is done through a technique called A/B testing, in which software automatically selects and randomizes participants into either the A group (control) or the B group (intervention), and serves each a different version of their product. Health care studies, on the other hand, often rely on research infrastructure that is separate from what patients and clinicians use during usual care, causing trials to be time-consuming, have high costs,^2^ and limited outcome generalizability.^3^ To address these issues, a new type of study, called a *Pragmatic Trial*, aims to perform medical research in the same setting as their intended target.^4^ Such designs have also been described as point-of-care trials,^5^ clinically integrated randomized trials,^2^ or learning healthcare system^5^ and aim to improve health care by embedding research within clinical care.

### 1.3 Importance

To study medical interventions without disrupting usual care, pragmatic trials tend to use existing electronic medical record (EMR) systems.^6^ Unfortunately, most EMRs do not provide built-in patient randomization tools; hence, many institutions started using encounter identification (ID) or patient ID numbers for study group allocation. However, such a process has never been studied or shown to produce sufficiently random assignments.

In order to validate the use of encounter ID or patient ID for research studies, one must first quantify the method’s randomness and performance. One way would be to use procedures from the cryptographic industry. To standardize random number and pseudorandom number generator testing, the National Institute of Standards and Technology developed a software package called NIST Special Publication 800-22^7^ (NIST) that applies 15 statistical tests to assess the randomness/unpredictability of their output. Another way would be to perform A/A testing,^1^ which uses the same randomization method as A/B testing but without performing the intervention; if the randomization is appropriate, then the demographics and study outcomes should be identical in each group.

### 1.4 Goals of This Investigation

We designed a study that validates the use of patient-specific data created during usual care and an electronic medical records-based randomization method (EMRRM) to generate appropriate group allocations as assessed by NIST and A/A testing. To ensure generalizability, we tested this approach in 3 diverse health care settings. We hypothesize that encounter ID and patient ID-based EMRRM will pass NIST randomization testing and produce groups with similar demographics and clinical outcomes in the outpatient, inpatient, and emergency department settings as assessed using A/A testing.

## 2 Materials and Methods

### 2.1 Study Design and Setting

We conducted 3 retrospective, pragmatic, randomized studies of patients at a single, urban, academic, quaternary-care hospital system in North Texas. The hospital uses Epic Systems as its EMR. The study protocol was approved by the local institutional review board (Study ID: STU-2023-0863). Because the study was self-funded and no intervention was performed, the trial was not registered in ClinicalTrials.gov.

### 2.2 Selection of Participants

To assess the effectiveness of randomization, we identified 3 different patient populations (outpatient, inpatient, and emergency department) to whom future interventions might be applied. We examined patients treated in those settings between January 1, 2022 and December 31, 2022.

Substudy #1 (outpatient): all patients seen in outpatient clinics with a diagnosis of diabetes mellitus on their problem list, as identified using International Classification of Diseases (ICD) 10 codes of E08, E10, E11, and E13.

Substudy #2 (inpatient): all patients admitted to the hospital with a diagnosis of pneumonia as identified using ICD 10 codes of J15, J16, J17, and J18.

Substudy #3 (emergency department): all patients seen emergency department with chest pain, as identified using ICD 10 code of R07.

### 2.3 Intervention

At our institution, an encounter ID is a unique 9-integer identifier specific to each individual and each health care encounter. It differs from the patient ID, which is also made of integers and is unique to each patient but stays the same for all their encounters. A Hash Function is a mathematical transformation method that takes in 1 number (key) and outputs a different number (hash) based on prespecified steps. EMRRM uses the mathematical Modulo (MOD 2) Hash Function that uses the encounter ID or patient ID as keys and checks them for parity. We assigned even hash outputs to study “Group 0” and odd hashes to study “Group 1.”

### 2.4 Outcome Measures

The outcome of this study was the EMRRM’s performance on the NIST test suite when using encounter ID-based and patient ID-based randomization. We also assessed EMRRM’s ability to generate demographically similar groups within each substudy population by comparing their age, sex at birth, race, comorbid medical conditions, and social history. Because previous epidemiological studies identified many unique medical conditions as confounders for each of the 3 studies, we chose to compare the same comorbidities across the 3 studies instead of making this comparison unnecessarily complicated. Because this is not an outcome study, this decision should have minimal impact on the results. We chose to report on the prevalence of the following medical diagnoses: diabetes, hyperlipidemia, hypertension, hypothyroid, obesity, reflux, and vitamin D deficiency.

For individual studies, additional outcomes were assessed using the following data points: Substudy #1 (outpatient) average glycemic control as measured by the last recorded hemoglobin A1c level at the time of clinic visit; substudy #2 (inpatient) hospital length of stay as defined by the difference between discharge and admission times; and substudy #3 (emergency department) admission rate as defined by the disposition order in EMR. In the case of randomization based on encounter ID, multiple participations of the same patient in the study were permitted. However, for randomization that was based on patient ID, each patient was allowed a single participation, and any subsequent eligible encounters were disregarded.

### 2.5 Data Collection and Processing

Data were abstracted from the Data Warehouse i2b2 platform by an institution-assigned analyst and included the following information: patient ID, encounter ID, age, sex at birth, race, comorbid medical conditions, social history, hemoglobin A1c level, hospital length of stay, and emergency department disposition. Data cleaning and analysis were performed using Python (Python Software Foundation)^8^ and open-source Python packages, including NumPy^9^ and Pandas.^10^

### 2.6 Data Analysis

In order to have sufficient data for the NIST package to perform all of its tests, we would require at least 1,028,016 patients for the overlapping template matching test. Because our entire health system does not have enough patients to satisfy this requirement, we instead chose an arbitrary length of 12 months for data collection for each substudy. Within each study, categoric data were compared using Fisher exact tests and χ^2^ tests. Continuous parametric data were compared using Welch’s *t* test, and nonparametric data were compared using the Mann-Whitney U test. Statistical analysis was performed using SciPy^11^ and Statsmodels^12^ Python packages. Randomization assessment was performed using the NIST-800-22^3^ statistical package and the NistRng^13^ Python package.

## 3 Results

### 3.1 Randomization Validation Results

EMRRM was used on 11,859 (5855 + 6004) encounters of 6214 (3073 + 3141) patients in the outpatient setting, 8141 (4071 + 4070) encounters of 3879 (1942 + 1937) patients in the inpatient setting, and 4127 (2070 + 2057) encounters of 3564 (1798 + 1766) patients in the emergency department setting. Table 1 compares encounter ID and patient ID-based EMRRM performance on individual statistical tests within the NIST 800-22 statistical package. This assessment was performed on randomizations performed in all 3 outpatient, inpatient, and emergency department settings. Per cryptographic standards, any test that achieves a *P* value of ≥.01 is considered passed. Except for 4 tests for which this study was underpowered (indicated by N/A in the table), both randomization methods passed either 10 or 11 out of 11 tests in each of the 3 substudies.

**Table 1:** Encounter ID and patient ID-based EMRRM performance on individual test components within the NIST 800-22 statistical package in the outpatient, inpatient, and the emergency department settings.

	Outpatient		Inpatient		Emergency department	
	Encounter ID	Patient ID	Encounter ID	Patient ID	Encounter ID	Patient ID
Frequency (monobit) test	0.171^a^	0.588^a^	0.991^a^	0.165^a^	0.839^a^	0.156^a^
Frequency test within a block	0.744^a^	0.018^a^	0.576^a^	0.613^a^	0.592^a^	0.625^a^
The runs test	0.053^a^	0.552^a^	0.357^a^	0.244^a^	0.078^a^	0.767^a^
Test for the longest-run-of-ones in a block	0.096^a^	0.047^a^	0.878^a^	0.119^a^	0.876^a^	0.229^a^
Binary matrix rank test	N/A^c^	N/A^c^	N/A^c^	N/A^c^	N/A^c^	N/A^c^
Discrete fourier-transform (spectral) test	0.449^a^	0.002^b^	0.358^a^	0.686^a^	0.110^a^	0.460^a^
Nonoverlapping template matching test	0.999^a^	1.000^a^	0.984^a^	0.918^a^	0.999^a^	0.984^a^
Overlapping template matching test	N/A^c^	N/A^c^	N/A^c^	N/A^c^	N/A^c^	N/A^c^
Maurer’s “Universal Statistical” test	N/A^c^	N/A^c^	N/A^c^	N/A^c^	N/A^c^	N/A^c^
Linear complexity test	N/A^c^	N/A^c^	N/A^c^	N/A^c^	N/A^c^	N/A^c^
Serial test	0.208^a^	0.340^a^	0.530^a^	0.308^a^	0.164^a^	0.739^a^
Approximate entropy test	0.208^a^	0.697^a^	0.757^a^	0.322^a^	0.175^a^	0.746^a^
Cumulative sums (Cusums) test	0.246^a^	0.555^a^	0.693^a^	0.163^a^	0.834^a^	0.123^a^
Random excursions test	0.001^b^	0.234^a^	0.106^a^	0.000^b^	0.105^a^	0.320^a^
Random excursions variant test	0.048^a^	0.386^a^	0.031^a^	0.046^a^	0.141^a^	0.468^a^

ID, identification; N/A, not applicable.

See Supplementary Appendix 1 for a description of each test.

Study demographics and substudy results.

a Passing score (*P* value ≥.01).

b Failing score (*P* value <.01).

c Study is underpowered to perform the test (*P* value is Not Applicable – N/A).

Table 2, Table 3, Table 4 demonstrate each substudy’s performance on the A/A test. As the tables indicate, group 0 and group 1 patient demographics appear similar when using the encounter ID-based and patient ID-based EMRRM. In the outpatient setting, only 1 demographic criterion in the encounter ID-based EMRRM showed a statistical difference (*P* value < .05), compared with 2 in the inpatient setting (1 in encounter ID and 1 in patient ID), and 2 in the emergency department setting (both in the patient ID-based EMRRM). Additionally, study-specific outcomes showed statistically similar HgbA1C, hospital length of stay, and emergency department chest pain dispositions between group 0 and group 1 in both the encounter ID-based or patient ID-based EMRRM methods. Because the encounter ID-based EMRRM allowed multiple participations of the same patient whereas patient ID-based EMRRM did not, the absolute numbers in each substudy differed between the 2 methods.

**Table 2:** A/A testing of outpatient diabetes glycemic control substudy.

		Encounter ID-based EMRRM			Patient ID-based EMRRM		
Category		Group 0	Group 1	*P* value	Group 0	Group 1	*P* value
Total	Count	5855	6004		3073	3141	
Sex	Female (%)	3094 (52.8)	3266 (54.4)	.093	1615 (52.6)	1727 (55.0)	.058
Age	In years (SD)	63.5 (12.6)	63.7 (12.5)	.376	63.0 (12.9)	62.9 (12.8)	.880
Ethnicity	Hispanic (%)	1217 (20.8)	1202 (20.0)		579 (18.8)	584 (18.6)	
	Non-Hispanic (%)	4274 (73.0)	4493 (74.8)		2290 (74.5)	2387 (76.0)	
	Unknown (%)	364 (6.2)	309 (5.1)	.017^a^	204 (6.6)	170 (5.4)	.112
Race	White (%)	2894 (49.4)	2949 (49.1)		1507 (49.0)	1551 (49.4)	
	Black or African American (%)	1747 (29.8)	1847 (30.8)		895 (29.1)	973 (31.0)	
	Asian (%)	409 (7.0)	429 (7.1)		235 (7.6)	221 (7.0)	
	American Indian or Alaska Native (%)	29 (0.5)	43 (0.7)		14 (0.5)	22 (0.7)	
	Native Hawaiian or Other Pacific Islander (%)	13 (0.2)	10 (0.2)		8 (0.3)	7 (0.2)	
	Unknown (%)	763 (13.0)	726 (12.1)	.312	414 (13.5)	367 (11.7)	.143
Comorbid Conditions	Diabetes (%)	5855 (100.0)	6004 (100.0)	1.000	3073 (100.0)	3141 (100.0)	1.000
	Hyperlipidemia (%)	2420 (41.3)	2503 (41.7)	.696	1394 (45.4)	1444 (46.0)	.647
	Hypertension (%)	3478 (59.4)	3631 (60.5)	.238	1880 (61.2)	1962 (62.5)	.308
	Hypothyroid (%)	531 (9.1)	587 (9.8)	.198	298 (9.7)	345 (11.0)	.104
	Obesity (%)	548 (9.4)	561 (9.3)	1.000	346 (11.3)	335 (10.7)	.465
	Reflux (%)	342 (5.8)	382 (6.4)	.250	207 (6.7)	246 (7.8)	.097
	Vitamin D deficiency (%)	499 (8.5)	507 (8.4)	.895	318 (10.3)	308 (9.8)	.500
Alcohol	Yes (%)	2165 (37.0)	2196 (36.6)		1180 (38.4)	1199 (38.2)	
	No (%)	3658 (62.5)	3783 (63.0)		1867 (60.8)	1927 (61.4)	
	Unknown (%)	32 (0.5)	25 (0.4)	.520	26 (0.8)	15 (0.5)	.191
Smoking	Current smoker (%)	401 (6.8)	393 (6.5)		223 (7.3)	198 (6.3)	
	Former smoker (%)	1781 (30.4)	1927 (32.1)		895 (29.1)	979 (31.2)	
	Never smoker (%)	3665 (62.6)	3671 (61.1)		1948 (63.4)	1953 (62.2)	
	Unknown (%)	8 (0.1)	13 (0.2)	.161	7 (0.2)	11 (0.4)	.145
HgbA1C	Mean (SD)	7.43 (1.7)	7.45 (1.8)	.516	7.338 (1.7)	7.331 (1.7)	.888

%, percent of total; EMRRM, electronic medical records-based randomization method; ID identification.

a Value signifies failed A/A test with *P* value <.05.

**Table 3:** A/A testing of inpatient pneumonia length of stay substudy.

		Encounter ID-based EMRRM			Patient ID-based EMRRM		
Category		Group 0	Group 1	*P* value	Group 0	Group 1	*P* value
Total	Count	4071	4070		1942	1937	
Sex	Female (%)	1977 (48.6)	1997 (49.1)	.666	947 (48.8)	959 (49.5)	.666
Age	In y (SD)	60.7 (18.1)	60.6 (17.8)	.873	62.4 (17.5)	61.9 (17.7)	.468
Ethnicity	Hispanic (%)	521 (12.8)	506 (12.4)		262 (13.5)	284 (14.7)	
	Non-Hispanic (%)	3489 (85.7)	3501 (86.0)		1644 (84.7)	1619 (83.6)	
	Unknown (%)	61 (1.5)	63 (1.5)	.873	36 (1.9)	34 (1.8)	.569
Race	White (%)	2575 (63.3)	2589 (63.6)		1259 (64.8)	1257 (64.9)	
	Black or African American (%)	1096 (26.9)	1095 (26.9)		470 (24.2)	484 (25.0)	
	Asian (%)	105 (2.6)	120 (2.9)		56 (2.9)	56 (2.9)	
	American Indian or Alaska Native (%)	9 (0.2)	15 (0.4)		4 (0.2)	6 (0.3)	
	Native Hawaiian or Other Pacific Islander (%)	2 (0.0)	6 (0.1)		2 (0.1)	2 (0.1)	
	Unknown	284 (7.0)	245 (6.0)	.192	151 (7.8)	132 (6.8)	.866
Comorbid conditions	Diabetes (%)	1627 (40.0)	1639 (40.3)	.786	777 (40.0)	752 (38.8)	.450
	Hyperlipidemia (%)	1986 (48.8)	1979 (48.6)	.894	965 (49.7)	968 (50.0)	.872
	Hypertension (%)	2312 (56.8)	2235 (54.9)	.090	1134 (58.4)	1089 (56.2)	.173
	Hypothyroid (%)	901 (22.1)	874 (21.5)	.485	420 (21.6)	419 (21.6)	1.000
	Obesity (%)	602 (14.8)	594 (14.6)	.827	310 (16.0)	288 (14.9)	.351
	Reflux (%)	1262 (31.0)	1283 (31.5)	.616	592 (30.5)	587 (30.3)	.917
	Vitamin D deficiency (%)	331 (8.1)	308 (7.6)	.365	153 (7.9)	141 (7.3)	.505
Alcohol	Yes (%)	911 (22.4)	855 (21.0)		483 (24.9)	449 (23.2)	
	No (%)	3033 (74.5)	3033 (74.5)		1352 (69.6)	1340 (69.2)	
	Unknown (%)	127 (3.1)	182 (4.5)	.003^a^	107 (5.5)	148 (7.6)	.019^a^
Smoking	Current smoker (%)	289 (7.1)	302 (7.4)		156 (8.0)	159 (8.2)	
	Former smoker (%)	1544 (37.9)	1597 (39.2)		699 (36.0)	720 (37.2)	
	Never smoker (%)	2135 (52.4)	2042 (50.2)		991 (51.0)	944 (48.7)	
	Unknown (%)	103 (2.5)	129 (3.2)	.104	96 (4.9)	114 (5.9)	.389
Length of stay (LOS)	Median in hours (IQR)	149.0 (192.4)	149.4 (188.7)	.758	143.9 (189.1)	142.7 (195.4)	.793

%, percent of total; EMRRM, electronic medical records-based randomization method; ID identification.

a Values signify failed A/A tests with *P* value <.05.

**Table 4:** A/A testing of ED chest pain admission rate substudy.

		Encounter ID-based EMRRM			Patient ID-based EMRRM		
Category		Group 0	Group 1	*P* value	Group 0	Group 1	*P* value
Total	Count	2070	2057		1798	1766	
Sex	Female (%)	1229 (59.4)	1259 (61.2)	.239	1068 (59.4)	1085 (61.4)	.226
Age	In y (SD)	51.2 (17.4)	50.8 (17.6)	.507	51.4 (17.5)	50.8 (17.8)	.294
Ethnicity	Hispanic (%)	412 (19.9)	439 (21.3)		357 (19.9)	374 (21.2)	
	Non-Hispanic (%)	1584 (76.5)	1552 (75.4)		1376 (76.5)	1329 (75.3)	
	Unknown (%)	74 (3.6)	66 (3.2)	.449	65 (3.6)	63 (3.6)	.620
Race	White (%)	997 (48.2)	953 (46.3)		875 (48.7)	839 (47.5)	
	Black or African American (%)	799 (38.6)	794 (38.6)		679 (37.8)	651 (36.9)	
	Asian (%)	57 (2.8)	51 (2.5)		53 (2.9)	46 (2.6)	
	American Indian or Alaska Native (%)	11 (0.5)	16 (0.8)		10 (0.6)	14 (0.8)	
	Native Hawaiian or Other Pacific Islander (%)	3 (0.1)	2 (0.1)		2 (0.1)	1 (0.1)	
	Unknown (%)	203 (9.8)	241 (11.7)	.339	179 (10.0)	215 (12.2)	.322
Comorbid conditions	Diabetes (%)	389 (18.8)	342 (16.6)	.073	342 (19.0)	283 (16.0)	.020^a^
	Hyperlipidemia (%)	240 (11.6)	237 (11.5)	.961	214 (11.9)	200 (11.3)	.601
	Hypertension (%)	826 (39.9)	762 (37.0)	.063	727 (40.4)	640 (36.2)	.011^a^
	Hypothyroid (%)	70 (3.4)	63 (3.1)	.597	63 (3.5)	53 (3.0)	.450
	Obesity (%)	70 (3.4)	81 (3.9)	.362	62 (3.4)	61 (3.5)	1.000
	Reflux (%)	317 (15.3)	311 (15.1)	.863	276 (15.4)	264 (14.9)	.744
	Vitamin D deficiency (%)	34 (1.6)	49 (2.4)	.097	30 (1.7)	42 (2.4)	.153
Alcohol	Yes (%)	586 (28.3)	555 (27.0)		510 (28.4)	489 (27.7)	
	No (%)	1181 (57.1)	1197 (58.2)		1004 (55.8)	990 (56.1)	
	Unknown (%)	303 (14.6)	305 (14.8)	.633	284 (15.8)	287 (16.3)	.875
Smoking	Current (%)	223 (10.8)	215 (10.5)		183 (10.2)	171 (9.7)	
	Former (%)	429 (20.7)	431 (21.0)		359 (20.0)	342 (19.4)	
	Never (%)	1146 (55.4)	1137 (55.3)		1002 (55.7)	996 (56.4)	
	Unknown (%)	272 (13.1)	274 (13.3)	.985	254 (14.1)	257 (14.6)	.904
Disposition	Admitted (%)	480 (23.2)	485 (23.6)		415 (23.1)	398 (22.5)	
	Discharged (%)	1361 (65.7)	1357 (66.0)		1191 (66.2)	1180 (66.8)	
	AMA/eloped/LWBS (%)	229 (11.1)	215 (10.5)	.806	192 (10.7)	188 (10.6)	.923

%, percent of total; AMA, patients that left Against Medical Advice; Eloped, patients that left without notifying staff; EMRRM, electronic medical records-based randomization method; ID identification; LWBS, patients that Left Without Being Seen.

a Values signify failed A/A tests with *P* value <.05.

## 4 Limitations

First, encounter ID and patient ID-based randomization did not pass all 15 tests in the NIST package, as 4 of those tests could not be completed due to insufficient randomization events. Because NIST-800-22 was designed to test random number generators that can output additional observations on as-needed bases, our data sets were far short of the 37,888 required by the binary matrix rank test, 1,028,016 required by the overlapping template matching test, 387,840 required by the Maurer’s “Universal Statistical” test, and 1,000,000 required by the linear complexity test. The failures on the random excursions test and the discrete fourier-transform (spectral) test can either be attributed to the method’s weakness or simply to a type I error as each of these tests was passed in other substudies. Additionally, by statistical definition, no random number generator can pass every test every time.

Second, EMRRM might be less effective in smaller health care systems. Because EMRRM relies on encounter IDs and patient IDs to generate random group assignments, health care systems with fewer yearly encounters or fewer patients will have a smaller range of encounter IDs and patient IDs and, therefore, less randomness. Our institution manages over 9 million patients and had over 20 million encounters in 2022—it is unclear if EMRRM can produce valid randomization results in smaller health care systems.

Third, regarding study-specific outcomes, group 0 and group 1 had both quantitatively and statistically similar results within each EMRRM method but were different when comparing the 2 EMRRM methods together. This, however, is not a valid comparison as the repeat patient enrollments in the encounter ID-based EMRRM did not occur in the patient ID-based EMRRM. Encounter ID-based EMRRM was therefore more likely to have the sicker patient bias as, for example, patients with higher HgbA1C were more likely to get a second clinic visit during the study period than those with better-controlled diabetes—this increased the number of higher HgbA1C data points in the encounter ID-based EMRRM but not in the patient ID-based EMRRM.

Finally, it is important to note that EMRRM is not immune to group unmasking once the Hash Function is discovered. This unmasking could lead to bias in the study, as most people can quickly determine whether a number is odd/even, regardless of its size. However, using more sophisticated Hash Functions, such as applying the cryptographic MD5 Hash Function prior to the MOD 2 used in this study, also resulted in good randomization (passed 10 out of 11 NIST tests) and would improve allocation concealment. Therefore, EMRRM should work appropriately as long as the Hash Function passes NIST testing and is disguised from patients and clinicians.

## 5 Discussion

The design of this trial is unique in that it is both a retrospective cohort study and a retrospective randomized control trial if 1 considers the randomization itself as the intervention and the performance on randomization testing as the outcome. The procedure for this study was first to generate the hypothesis (before gathering the data), then randomize the patients (intervention), and finally analyze the randomization performance (outcome). We hypothesized that because no intervention was performed, the outcomes between groups in each substudy should have been the same when performing A/A testing. If the randomization were biased or inadequate, A/A testing would have shown a false difference between the groups. Although this trial was done retrospectively, it could have easily been done prospectively and yielded the same results.

Based on NIST and A/A testing, both encounter ID-based and patient ID-based EMRRM methods appear to randomly generate similar study groups that meet cryptographic standards. Because neither encounter ID nor patient ID relies on patient-specific data (ie, name or birthday), they cannot be predicted or biased by patient demographics. Encounter IDs are generated sequentially at the health system level and assigned to all patient encounters, including hospital admissions, office visits, telemedicine, procedure visits, allied health/nurse visits, patient messages, home care, laboratory, radiology, and pharmacy and medication management encounters. Because encounter IDs are assigned at the time of patient contact/scheduling rather than during the actual encounter, patients with later appointments can have much smaller encounter IDs than those evaluated earlier. All of the random factors influencing the timing of a patient initiating an encounter add unpredictability to the encounter ID generation process. At our health system, with new encounter IDs being assigned at a rate of 90 per minute, the scheduling staff answering a phone call after 2 rings instead of one may alter the encounter ID by several digits. It is, therefore, not surprising that encounter ID-based randomization did well on the NIST analysis. Interestingly, similar factors impact patient ID generation, which is why patient ID-based randomization also did well on the NIST evaluation.

Aside from the ease of use, EMRRM has built-in quality assurance and integrity checks for clinical research projects: knowing the token (ie, encounter ID or patient ID) and the Hash Function allows anyone to recalculate the research group assignment retrospectively. This allows for a much easier intention-to-treat and per-protocol analysis, as both correct group assignments and received interventions can easily be compared. This same property also reduces the chance of group assignment falsification or manipulation. Because encounter ID and patient ID are unalterable and automatically stored within the EMR, it reduces the technological overhead and need for external data storage and privacy or security protection. Additionally, EMRs with clinical decision support infrastructure can use the ID variables to automatically initiate experimental actions, such as automatically documenting emergency department medications for improved medical coding.^14^

Unlike traditional clinical trials that study medical interventions in carefully controlled settings and study populations, pragmatic trials excel at answering more practical questions. In order for pragmatic trials to yield unbiased results, they need a convenient and valid way to perform randomization. Although many institutions have been using encounter and patient-based information for research management purposes, there are no written guidelines or recommendations on their use for randomization.^6^ This study adds value by validating the use of encounter ID and patient ID-based EMRRMs for use in pragmatic studies.

In conclusion, the quality of group assignments in clinical trials is critical to establishing a cause-and-effect relationship. In pragmatic trials, an ideal randomization procedure results in fair group assignments that can neither be manipulated nor predicted, and can easily be embedded into the standard clinical workflow. Because encounter ID and patient ID-based EMRRM works seamlessly with most healthcare workflows and have low entry costs due to its use of existing technological infrastructure, it can be used to complement current research infrastructure and improve research productivity at most healthcare institutions. The next step would be to externally validate these findings.

## Author Contributions

JF and SAM conceived the study. JF, JR, RT, EO, BT, and SAM developed the methodology. JF, JR, and BT performed the analysis. BT provided statistical advice. JF drafted the manuscript, and all authors contributed substantially to its revision. JF takes responsibility for the paper as a whole.

## Funding and Support

This study was self-funded by the University of Texas Southwestern Medical Center Department of Emergency Medicine.

## Conflict of Interest

All authors have affirmed they have no conflicts of interest to declare.

## References

[1] Kohavi R., Longbotham R., Sammut C., Webb G.I. (2017). Encyclopedia of Machine Learning and Data Mining.

[2] Esteller-Cucala M., Fernandez V., Villuendas D. (2020). Evaluating personalization: the AB testing pitfalls companies might not be aware of-a spotlight on the automotive sector websites. Front Artif Intell.

[3] Rukhin A, Soto J, Nechvatal J, et al. A Statistical Test Suite for Random and Pseudorandom Number Generators for Cryptographic Applications.

[4] Vickers A.J., Scardino P.T. (2009). The clinically-integrated randomized trial: proposed novel method for conducting large trials at low cost. Trials.

[5] Van Spall H.G.C., Toren A., Kiss A., Fowler R.A. (2007). Eligibility criteria of randomized controlled trials published in high-impact general medical journals: a systematic sampling review. JAMA.

[6] Ford I., Norrie J. (2016). Pragmatic trials. N Engl J Med.

[7] Fiore L.D., Brophy M., Ferguson R.E. (2011). A point-of-care clinical trial comparing insulin administered using a sliding scale versus a weight-based regimen. Clin Trials.

[8] van Rossum G. (1995).

[9] Van Der Walt S., Colbert S.C., Varoquaux G. (2011). The NumPy array: a structure for efficient numerical computation. Comput Sci Eng.

[10] McKinney W. (2010). SciPy Proceedings.

[11] Virtanen P., Gommers R., Oliphant T.E. (2020). SciPy 1.0: fundamental algorithms for scientific computing in Python. Nat Methods.

[12] Seabold S., Perktold J. (2010). SciPy Proceedings.

[13] Pasqualini L. Random Number Generator NIST Test Suite Framework for Python 3.6 - SAILab. https://github.com/InsaneMonster/NistRng.

[14] Furmaga J., Courtney D.M., Lehmann C.U. (2022). Improving emergency department documentation with noninterruptive clinical decision support: an open-label, randomized clinical efficacy trial. Acad Emerg Med.

