# Supplementary material for: Use of Patient-Specific Information for Randomization in Clinical Research: A Randomized Trial
Source: J Am Coll Emerg Physicians Open. 2025 Jun 30;6(4):100215. doi: 10.1016/j.acepjo.2025.100215 (PMC12269581; doi:10.1016/j.acepjo.2025.100215)
Supplement: Supplementary Appendix 1 [file mmc1.docx]

**Appendix A: Description of individual tests used in the NIST 800-22 statistical package:**

NIST 800-22 randomness testing requires a sequence made up only of *zeros* and *ones*. By applying Modulo-2 to either patient ID or encounter ID, we obtain *zero* if the ID number is even and *one* if the ID is odd. When employing this technique on patients in the order they presented for the study, we obtain a sequence of *zeros* and *ones* that can be used as input for the following tests:

- **Frequency (Monobit) Test:** assesses the proportion of zeros and ones in the entire sequence to determine if they are approximately equal, as would be expected in a truly random sequence.
- **Frequency Test within a Block:** assesses the proportion of zeros and ones within M-bit blocks to check if the frequency of ones is approximately M/2, as would be expected in a truly random sequence.
- **The Runs Test:** examines the runs (uninterrupted sequences of identical bits of zeros or ones) in the entire sequence to see if their number and lengths are as expected for a random sequence.
- **Test for the Longest-Run-of-Ones In A Block:** determines if the length of the longest run of ones within the tested sequence (M-bit block) is consistent with what would be expected in a random sequence.
- **Binary Matrix Rank Test:** checks for linear dependence among fixed-length substrings of the original sequence by evaluating the rank of disjoint sub-matrices.
- **Discrete Fourier Transform (Spectral) Test:** detects periodic repetitive patterns that are near each other in the tested sequence, that would be different from what would be expected in a random sequence.
- **Non-Overlapping Template Matching Test:** counts the number of occurrences of pre-defined target substrings (templates) in the sequence without overlapping.
- **Overlapping Template Matching Test:** counts the number of occurrences of pre-defined target substrings (templates) in the sequence but allows for overlapping.
- **Maurer’s “Universal Statistical” Test:** measures the compressibility of the sequence without loss of information. Sequences that allow significant compressibility indicate redundancy and are therefore considered non-random.
- **Linear Complexity Test:** assesses the complexity of the sequence by determining the length of a linear feedback shift register (LFSR) that can generate the sequence. Short LFSRs imply non-randomness.
- **Serial Test:** evaluates the frequency of all possible overlapping M-bit patterns across the entire sequence to check if uniformly distributed.
- **Approximate Entropy Test:** compares the frequency of overlapping blocks of two consecutive lengths (m and m+1) to detect patterns and regularities.
- **Cumulative Sums (Cusums) Test:** each group is assigned either -1 or +1, with the test being the cumulative sum of the partial sequences. The result is the maximum excursion from zero, which should not be too erratic or too smooth.
- **Random Excursions Test:** examines the number of cycles between cumulative sum reaching zero to identify deviations from randomness.
- **Random Excursions Variant Test:** similar to the Random Excursions Test but focuses on the number of visits to each state (i.e. cumulative sum of -9, -8, …, -1 and +1, +2, …, +9)
